# Additional Intraoperative Autologous-Derived Platelet-Rich Stroma to Transanal Flap Repair for the Treatment of Cryptoglandular Transsphincteric Fistulas in a Tertiary Referral Center: Long-Term Outcomes of a Prospective Pilot Study

**DOI:** 10.3390/bioengineering12020105

**Published:** 2025-01-23

**Authors:** Michiel T. J. Bak, Caroline D. M. Witjes, Roy S. Dwarkasing, Jeanine H. C. Arkenbosch, W. Rudolph Schouten, Jochem C. van Veen, Joris A. van Dongen, Gwenny M. Fuhler, C. Janneke van der Woude, Annemarie C. de Vries, Oddeke van Ruler

**Affiliations:** 1 Department of Gastroenterology and Hepatology, Erasmus University Medical Center, 3015 GD Rotterdam, The Netherlands; m.bak@erasmusmc.nl (M.T.J.B.);; 2 Department of Surgery, IJsselland Hospital, 2906 ZC Capelle aan den Ijssel, The Netherlands; 3 Department of Surgery, Erasmus University Medical Center, 3015 GD Rotterdam, The Netherlands; 4 Department of Radiology, Erasmus University Medical Center, 3015 GD Rotterdam, The Netherlands; 5 Department of Plastic, Reconstructive and Hand Surgery, University Medical Center Utrecht, Utrecht University, 3584 CX Utrecht, The Netherlands

**Keywords:** perianal fistula, platelet-rich stroma, stromal vascular fraction, platelet-rich plasma, autologous, cell therapy

## Abstract

Transanal advancement flap repair (TAFR) fails in approximately 30–40% of patients with a cryptoglandular transsphincteric fistula. An additional intraoperative injection of autologous platelet-rich stroma (PRS) with TAFR proved to be safe, feasible, and effective in the short term for the treatment of cryptoglandular transsphincteric fistula in a tertiary referral center. In this study, we assessed the long-term outcomes in patients with a cryptoglandular transsphincteric fistula who were treated with an additional intraoperative autologous PRS injection with TAFR (n = 43). The majority of the patients (88%) had a complex transsphincteric fistula (high transsphincteric and/or multiple side tracts) and underwent (one or more) fistula procedure(s) aimed at fistula repair (56%) before study inclusion. At a median follow-up time of 4.2 years [IQR 3.5–5.1], long-term primary clinical closure (i.e., clinical closure of the treated external fistula opening(s) after TAFR with additional PRS injection without the need for any re-interventions during long-term follow-up) was observed in 77% of the patients. Subsequently, 94% of these patients also reached radiological healing (i.e., fibrotic fistula tract on MRI). Recurrence after clinical closure or radiological healing was observed in 9% and 5%. Unplanned re-interventions were performed in 12% of the patients for recurrent or residual fistulizing disease. In this uncontrolled pilot study, additional autologous PRS injection with TAFR showed promising outcomes, as long-term primary clinical closure and, subsequently, radiological healing was reached in the vast majority of tertiary referral patients with a (complex) cryptoglandular transsphincteric fistula at long-term follow-up. In addition, recurrence rates were low. Future randomized research is warranted to study the effects of PRS.

## 1. Introduction

Transanal advancement flap repair (TAFR) is one of the oldest surgical sphincter-preserving procedures and is often performed for the treatment of cryptoglandular transsphincteric fistulas. Despite many attempts to improve the outcomes of TAFR, this procedure fails in approximately 30–40% of patients with cryptoglandular transsphincteric fistulas [1,2,3]. In addition, a weighted recurrence rate of 25% has been reported [4]. The probable reason for failure is the ongoing chronic inflammation in the remaining fistulous tract, induced by pro-inflammatory mediators and cytokines [5,6,7]. Based on these findings, the suppression of chronic inflammation may improve the outcomes of TAFR.

Stromal vascular fraction (SVF), obtained from subcutaneous lipoaspirate through mechanical fractionation, contains a heterogeneous population of cells including macrophages, leucocytes, fibroblasts, endothelial cells, pericytes, and adipose-derived stromal cells (ASCs), with preserved cell–cell connections, including the extracellular matrix [8]. SVF has both regenerative wound healing and immunomodulatory properties and has been shown to enhance clinical wound healing [9]. Platelet-rich plasma (PRP), an autologous product obtained from a venous blood sample, contains high concentrations of growth factors, which also promote wound healing and provide mechanical support [10].

A combined autologous product of SVF and PRP, referred to as platelet-rich stroma (PRS), is used as a treatment for several other medical conditions such as androgenetic alopecia [11]. An additional autologous injection of PRS was introduced in our center for both cryptoglandular transsphincteric fistulas and Crohn’s perianal fistulas (CPF) in a pilot setting [12,13]. Treatment with PRS, as an add-on to TAFR for cryptoglandular transsphincteric fistulas or in combination with closure of the internal orifice for CPF, proved to be safe and feasible in two pilot studies [12,13]. In the short term (<1 year), primary clinical closure (i.e., clinical closure after TAFR with additional PRS injection without the need for any re-interventions) was reached in 84% of tertiary referral patients with a cryptoglandular transsphincteric fistula. Of those who reached primary clinical closure, radiological healing was observed in 89% [12].

As knowledge of the long-term outcomes of this treatment is lacking, this study aimed to assess the long-term clinical and radiological outcomes in patients with a cryptoglandular transsphincteric fistula that was treated with TAFR and additional autologous PRS injection who were included in this pilot study in our tertiary referral center.

## 2. Materials and Methods

Between December 2017 and February 2020, consecutive adult patients with a cryptoglandular transsphincteric fistula who were scheduled for TAFR were enrolled in a prospective, non-randomized, pilot cohort study after providing informed consent in our tertiary referral center [12]. The exclusion criteria were associated pelvic abscess, rectovaginal fistula, the presence of a second internal orifice at or above the dentate line, a history of Crohn’s disease, immune-compromised status (e.g., active human immunodeficiency virus), hematological disorders, coagulation disorders, and/or any oncological event in the previous 5 years [12]. Patients who were diagnosed with Crohn’s disease during follow-up were excluded. All patients underwent standardized TAFR with an additional injection of autologous PRS, performed by three colorectal surgeons (W.R.S., O.v.R., and E.G.)

### 2.1. Operative Technique

The operative technique was described earlier in Schouten et al. [12]. A video of the procedure was previously published [14]. After induction of spinal or general endotracheal anesthesia, metronidazole (500 mg) and cefuroxime (2000 mg) were administered intravenously. Up to the discretion of the treating surgeon, a temporary drain or Malecot catheter was placed to improve drainage in cases where extensive side branches or cavitation was present. The external opening was cored out to the level of the external anal sphincter and left open to provide adequate drainage. If TAFR was considered feasible, lipoaspiration for the harvesting of the SVF was initiated. Due to the hypothetical risk of subcutaneous infection, liposuction was always performed prior to TAFR or by renewing the sterile field and operating team. A small paravertebral skin incision was made bilaterally approximately 5 cm cranial to the posterior superior iliac spine. The subcutaneous adipose tissue was infiltrated bilaterally with 40 mL of 0.9% saline solution, containing 20 mg/mL of lidocaine 2% with 1:100.000 adrenaline added to 500 mL of 0.9% saline solution. Using vacuum, 15 mL of lipoaspirate was harvested on either side with a double syringe (Arthrex GMBH, München, Germany). Hereafter, each double syringe was put in a sterile centrifuge bucket. During the first centrifugation cycle (5 min, 2500 rpm), the TAFR procedure was continued. A Lone Star retractor (Lone Star Medical Products^®^, Inc., Houston, TX, USA) was used to expose the internal orifice with the purpose of curating the internal orifice. The remains of the anal gland were curated or excised if possible. The internal orifice was then sutured with 2.0 or 3.0 vicryl suture. The base of the flap comprised approximately one-third of the circumference, consisting of mucosa, submucosa, and some of the most superficial fibers of the internal anal sphincter, to guarantee blood supply to the apex of the flap. Subsequently, the apex of the flap was raised from the level of the dentate line and mobilized over a distance of approximately four to five centimeters proximally. The flap was advanced and sutured over the closed internal orifice to the neo-dentate line with absorbable sutures 2.0 Monocryl (Ethicon, Inc., Somerville, NJ, USA). The obtained lipoaspirate was centrifuged (2500 rpm, 5 min) using the Rotofix 32A centrifuge (Andreas Hettich, GmbH & Co., KG, Tuttlingen, Germany), which resulted in three separated fractions: oil, condensed fatty tissue, and aqueous fraction. The oil and fluid fractions were removed from the lipoaspirate. Mechanical fractionation of the centrifuged lipoaspirate was performed by vigorously passing the lipoaspirate forwards and backward 30 times through a disposable one-hole fractionator (⌀1.4 mm, luer-to-luer transfer, Tulip). After mechanical fractionation, the fatty tissue was centrifuged (5 min, 2500 rpm). After the second centrifugation, the upper oily fraction was removed, resulting in 1 mL of SVF [15]. Simultaneously, 15 mL of whole blood was centrifuged (1500 rpm, 4 min), after which 4–5 mL of PRP was obtained from the upper layer (plasma) [16]. The SVF was combined with the PRP (i.e., PRS), with a volume of 6 mL, and was injected into the tissue surrounding the curated internal orifice within approximately 2 mm, as well as into all quadrants of the fistula wall along the fistula tract(s) in several micro-blebs The standard local postoperative protocol included the administration of postoperative antibiotics, discharge from the hospital after 3 days if possible, and no specific regulations for bed rest.

### 2.2. Follow-Up and Clinical Data Collection

Regular outpatient follow-up visits were scheduled for all patients up to 1 year postoperatively (6 weeks and 3–6–12 months postoperatively). In case clinical and/or radiological healing was achieved, patients were invited to contact and/or visit the outpatient clinic annually for up to 3 years postoperatively. In case clinical and/or radiological healing was not achieved within one year, further treatment and follow-up visits (including repeat MRI scans) were discussed by the treating surgeon on a case-by-case basis. In the outpatient clinic, a physical examination was conducted to assess the closure of the external orifice.

Data were collected from the individual electronic medical files at preoperative visits, at the time of fistula surgery, and postoperatively, including patient-related characteristics (e.g., age and smoking) and surgical characteristics (e.g., necessity for a re-intervention) up to the end of the study (October 2023).

### 2.3. MRI Evaluation

Prior to surgery, MRI was performed in all patients using a 1.5 T. system with a four-channel phased array pelvic coil. The field of view consisted of the lower pelvis, perineum, and skin area, with a full display of the anus and lower mid-rectum. The MRI protocol included T2-weighted (T2W) sequences in three planes: axial T2W with fat saturation, and sagittal and coronal T2W. Fistulas were identified based on hyperintense (white) signals. The course of the fistula tract, the presence of secondary tracts, and associated abscess(es) were examined. When the physical examination revealed closure of the external orifice (s), the MRI was repeated. Radiological healing of the fistula tract(s) was defined as the absence of hyperintense signals (i.e., a complete fibrotic fistula tract). An experienced radiologist (R.D.), blinded to the disease characteristics and outcomes, interpreted the anonymized images of the pre- and postoperative MRIs in a random order.

### 2.4. Outcomes

The primary outcome of this study was long-term primary clinical closure, defined as the complete closure of all treated external opening(s) at physical examination at the last documented follow-up visit following TAFR and PRS injection, without the need for any re-interventions. Secondary endpoints included (I) radiological healing, as described above, at the last available MRI, and (II) fistula recurrence, defined as the reopening of the external orifice after clinical and/or radiological healing. In addition, we reported the overall clinical and radiological healing rates of the study population and the need for unplanned re-interventions during long-term follow-up.

### 2.5. Statistical Analyses

Descriptive statistical analyses (frequency, percentage, mean, standard deviation [SD], median, and interquartile range [IQR]) were used to describe the research sample. Categorical variables were quoted as the number and percentage. Continuous variables were tested for normality using the Kolmogorov–Smirnov test. Normal distributed variables were presented as mean and SD, whilst non-normal distributed variables were presented as median and IQR. Data analysis was performed using SPSS (version 21, IBM, Chicago, IL, USA).

## 3. Results

During follow-up, 2 of the 45 patients, who were included in the earlier pilot study, were diagnosed with Crohn’s disease during long-term follow-up and were excluded from further analysis. Of the remaining patients (n = 43), the majority (70%) were male (Table 1). The mean age at surgery was 46.1 years (SD 12.0), and the median follow-up time was 4.2 years (IQR 3.5–5.1). The majority of the patients (88%) had a complex transsphincteric fistula and underwent (one or more) fistula procedure(s) aimed at fistula repair (56%) prior to study inclusion.

### 3.1. Short-Term Outcomes After TAFR and Additional PRS Injection

Within one year following TAFR and additional PRS injection, primary clinical closure was observed in 84% (36/43) of the patients [12]. A postoperative MRI was performed in 35/36 of the patients with primary clinical closure, while in 1 patient, pre- and postoperative imaging could not be obtained due to claustrophobia. Radiological healing was observed in 89% (31/35) of these patients.

### 3.2. Long-Term Clinical Outcomes After TAFR and Additional PRS Injection

**Figure 1:**
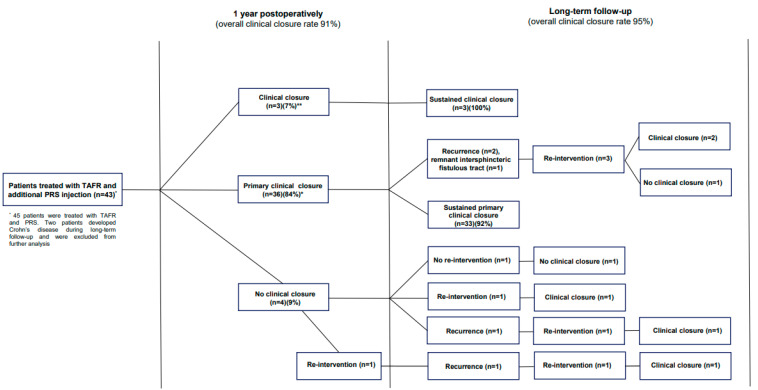
Flowchart of clinical outcomes following TAFR and additional PRS injection. **Abbreviations.** TAFR = transanal advancement flap repair; PRS = platelet rich stroma. * Primary clinical closure was defined as complete clinical closure <1 year without need for unplanned re-interventions. ** Clinical closure was defined as complete clinical closure <1 year after need for unplanned re-interventions.

Of those who reached primary clinical closure within one year (n = 36), long-term primary clinical closure was observed in 33 patients (92%), resulting in an overall long-term clinical closure healing rate of 77% (33/43) (Figure 1). Of the three patients in whom healing did not persist, two patients experienced recurrence, and a remnant intersphincteric fistulous tract was observed by imaging in one patient. All three patients underwent a surgical re-intervention with the aim of closure, after which clinical closure was observed in two patients. The patient with a remaining active fistula refrained from further treatment.

Of those who reached secondary clinical closure within one year (n = 3) (i.e., clinical closure after the need for unplanned re-interventions), sustained clinical closure was observed during long-term follow-up in all three patients. At the end of the follow-up, clinical closure was reached in 95% (n = 41) of the patients. The remaining patients (n = 2), who did not reach clinical closure, refrained from further treatment, as these patients were satisfied with their current status.

### 3.3. Long-Term Radiological Outcomes After TAFR and Additional PRS Injection

During the study period, at least one MRI was performed in 42 patients. During the long-term follow-up (>1 year postoperatively), a total of 27 MRIs were performed in 16/42 (38%) of the patients (median 1; IQR 1–2). The indication for additional imaging was conformation of clinical findings during follow-up (without a re-intervention) (n = 10, 37%), fistula-related complaints (n = 14, 52%), or postoperative monitoring response after a re-intervention (n = 3, 11%).

In those who reached long-term primary clinical closure and who underwent a postoperative MRI (32/33), complete radiological healing was observed in 30/32 patients (94%) (overall radiological healing rate after single-staged TAFR with additional PRS injection: 71%, 30/42).Of those who reached clinical closure and with an available MRI (n = 40), radiological healing at the last available MRI was observed in 35 patients (88%) (overall radiological healing rate: 78%, 35/42). Of those patients (n = 5) who achieved clinical closure but no radiological healing, one patient was lost to follow-up, two patients had a near-complete fibrotic fistula tract on MRI, and two patients did not undergo additional MRI following clinical closure.

### 3.4. Long-Term Recurrence After Clinical and Radiological Healing

In total, four patients (4/43, 9%) experienced recurrence after achieving clinical closure. All of these patients underwent a re-intervention. Subsequently, three out of four patients did reach clinical closure after the re-intervention. The remaining patient without clinical closure refrained from further treatment due to satisfaction with their current status. Two patients clinically experienced fistula recurrence after achieving radiological healing. As radiological healing was achieved in 37 patients during the entire study period, the recurrence rate following complete radiological healing was 5% (2/37).

### 3.5. Re-Interventions During Follow-Up

During the long-term follow-up (>1 year following TAFR and additional PRS injection), five patients (12%) underwent a total number of eight unplanned re-interventions for recurrent of residual fistulizing disease (incision and drainage [n = 3], seton placement [n = 1], additional PRS injection [n = 1], additional PRS injection and re-TAFR [n = 1], fistulotomy [n = 1], and laser [n = 1]) (Figure 1; Table 2).

## 4. Discussion

This uncontrolled pilot study showed that an additional autologous PRS injection with TAFR is a promising treatment for (complex) cryptoglandular transsphincteric fistulas and may improve the outcomes of TAFR. Clinical closure was reached in the vast majority (77%) of patients, who were treated in a tertiary referral center following single-stage TAFR with an additional PRS injection, without the need for any re-interventions after a median long-term follow-up of 4.2 years. Subsequently, complete radiological healing was reached in 94% of these clinically healed patients. In addition, we observed a high rate of overall long-term clinical closure (95%) and, subsequently, high radiological healing rates (88%) in the patients with clinical closure. In addition, recurrence rates were low.

An injection of autologous PRS may improve the outcomes of TAFR, as higher primary healing rates have been observed in this current study compared to our two most recent series, which described a one-year postoperative healing rate of between 59% and 68% following TAFR alone in a tertiary setting [1,2]. Furthermore, the outcomes are promising, as a further increase in tertiary referrals of patients with complex fistulas has been observed in our center over the past years, which is demonstrated by the high rates of complex transsphincteric fistulas (88%) and the higher rates of patients who underwent prior surgical procedures aimed at fistula repair in this current study (56%) compared to the most recent published series from our center (26%) [1]. In addition, lower recurrence rates after TAFR have been reported in this current study (8%) compared to a weighted recurrence rate of 25% [4]. Despite these promising results, future randomized research is warranted to assess the outcomes of additional autologous PRS injection with TAFR compared to TAFR alone.

The meta-analysis of Stelllingwerf et al. reported a weighted success rate of 75% for patients with cryptoglandular fistulas who were treated with TAFR [4]. However, these results should be interpreted carefully, as the healing rates ranged widely between the included studies (33–95%) due to the heterogeneity that was observed with regard to differences in outcome measurements, small sample sizes, and the follow-up times (I^2^ = 89%). Sixteen studies in this meta-analysis assessed the outcomes of flap repair in cryptoglandular fistulas. Of these studies, three did not define the success of TAFR, and four studies used a less stringent definition of treatment success (e.g., the absence of discharge). Furthermore, no data on re-interventions were presented [4]. Therefore, we believe that the weighted success rate of 74% cannot be extrapolated to a weighted primary clinical closure rate of 74%. As clinical closure after single-stage fistula surgery is essential to assess the outcome of a certain surgical technique, we used this stringent endpoint as the primary outcome in our studies [12].

As the persistent overall high failure rate of TAFR (30–40%) is likely due to the ongoing chronic inflammation in the remaining fistulous tract, suppression of this chronic inflammation process in order to generate wound healing seems to be the key to success [5,6,7]. Furthermore, the improved function of fibroblasts and endothelial cells caused by SVF may contribute to achieving wound healing [17,18]. The use of autologous PRS offers several advantages over the treatment with allogenic and autologously cultured or enzymatic ASC treatments, such as the significantly lower costs and lower burden of the carbon footprint due to the possibility of on-site preparation and the use of PRS during the surgical procedure, when compared to the laborious preparations for autologous or allogenic cultured or enzymatically obtained ASCs. Especially the use of mechanically fractioned SVF, similar to our study, seems to be beneficial in diseases that are characterized by a pro-inflammatory character such as arthritis or disturbed wound healing (e.g., a perianal fistula) [9]. SVF is thought to augment wound healing through the additional presence of a heterogeneous mixture of leukocytes, macrophages, ASCs, stromal, fibroblasts, and vascular endothelial cells, all embedded in a fibrovascular network. While mechanically isolated adipose-derived SVF retains the extracellular matrix (ECM), enzymatically isolated SVF contains a single-cell suspension of the aforementioned cells without an ECM [19]. ECM preservation has an essential role in the regenerative properties of SVF and provides structural support to cells such as ASCs [20]. ASCs reside as pericytes and supra-adventitial cells (precursor cell types) around vessels that are present in the extracellular matrix. In this way, the extracellular matrix most likely prevents ASCs from diffusion from the site of injection within the first hours [20,21]. PRP contains a high concentration of growth factors that promote wound healing and neovascularization [22]. Additionally, PRP stimulates ASCs to produce paracrine factors, which are relevant to achieving tissue repair [23]. Therefore, autologous PRS injection was introduced in our center to improve the outcomes of TAFR.

To date, only one pilot study, conducted by Tutino et al. (n = 9), assessed the outcomes of autologous SVF and PRP injection with additional closure of the internal orifice in patients with a complex transsphincteric fistula (i.e., a highly transsphincteric fistula or a transsphincteric fistula with a horseshoe extension) [24]. At the 1-year follow-up, clinical fistula closure was observed in 78% of the patients. These results are in line with our short-term results [12]. Unfortunately, postoperative imaging in this study was not obtained [24]. Several studies assessed the effects of treatment with autologous ASCs, in combination with PRP or microfat, for CPF [13,25,26]. A pilot study in our center showed that treatment with closure of the internal orifice in combination with PRS injection is safe and feasible in patients with treatment-refractory CPF (n = 25), resulting in a complete clinical closure rate of 52%, within one year postoperatively, and a radiological healing rate of 38% at a median of three months postoperatively [13]. The long-term outcomes of this pilot study are awaited. Serrero et al.(n = 10) reported a clinical closure rate of 77% at week 48 in patients with CPF who were treated with autologous SVF and microfat combined with closure of the internal orifice [25]. Another pilot study (n = 10) reported a 70% combined remission rate (defined as the complete cessation of fistula suppuration with no collection > 2 cm on MRI) at 3 years follow-up in therapy-refractory CPF patients (refractory to surgical drainage with setons and anti-TNF alpha treatment) following autologous SVF and microfat combined with closure of the internal orifice [26]. Only three studies have assessed the outcomes of mechanically fractioned SVF for the treatment of cryptoglandular fistulas [27,28,29]. Dalby et al. reported the short-term outcomes of SVF treatment in combination with closure of the internal orifice in patients with a cryptoglandular transsphincteric fistula. Clinical fistula closure and combined clinical fistula closure with MRI healing (the absence of collections >2 cm at the location of the former fistula) were reached in 51% and 48%, respectively [27]. A randomized controlled trial, comparing SVF treatment as an add-on to fistula surgery with fistula surgery alone, reported significantly higher clinical closure rates up to 3 months in favor of patients who were treated with additional SVF compared to the control group but with a similar persistent clinical closure rate at 6 months [28]. Finally, Borowski et al. reported the outcomes of seven patients with a cryptoglandular fistula who were treated with SVF in combination with closure of the internal orifice. At 6 months follow-up, five patients reached clinical closure (71%), and in four of these patients, sustained fistula closure was reported at a median follow-up of 46 months [29]. The outcomes of these studies, supported by the results of this current study, suggest that SVF alone or in combination with PRP or microfat is promising for the treatment of complex cryptoglandular or Crohn’s perianal fistula. However, larger studies (preferably randomized) are necessary to confirm these findings.

It is still unknown whether the addition of PRP to SVF leads to a potential increase in wound healing properties. A recently published meta-analysis, with data from 14 studies including 514 patients, reported an overall healing rate of 62.4% after PRP injection alone and 83.1% after local injection of PRP with additional surgical procedures. However, these results should be interpreted with caution due to the significant heterogeneity amongst the included studies (I^2^ = 60.1%; *p* < 0.05) [30]. Studies assessing the effects of PRP as an add-on to TAFR for the treatment of patients with a cryptoglandular fistula are limited. Van der Hagen et al. (n = 10) reported a 1-year clinical closure rate of 90% in patients who were treated with a TAFR combined with PRP injection. In their follow-up study, a healing rate of 83% at 2 years follow-up was reported [31,32]. The data from our center showed no additional effect of PRP as an addition to TAFR in both the short and long term compared to patients who were treated with TAFR alone in a large cohort of patients (n = 219) [1]. However, the methodology (i.e., preparation of the product[s]) and endpoints differ between the studies, assessing the effects of SVF (with or without PRP) or PRP alone, which hampers the comparison of outcomes between these studies [13,25,26,27,28,29]. Standardized treatments and endpoints in clinical trials are warranted to improve the quality of the treatment and to compare outcomes between studies.

To the best of our knowledge, the present study is the first study that evaluates the effect of SVF that is enriched with PRP as an adjunct to TAFR for cryptoglandular fistulas during long-term follow-up. However, this study has several limitations, of which the limited sample size, absence of a control group, and study location (i.e., a tertiary referral center) can be considered the main limitations. Despite the promising results in this pilot study, randomized controlled trials are warranted to study the effects and the positioning of the additional PRS injection in the treatment of cryptoglandular transsphincteric fistulas. In addition, no patient-reported outcomes (e.g., quality of life and incontinence) were routinely assessed in a structured manner and could, therefore, not be reported as an endpoint. Furthermore, the long-term follow-up (>1 year following treatment) was not standardized but left to the discretion of the treating physician and the patient’s needs. For instance, an additional MRI during long-term follow-up was only performed in 38%. However, these outcomes reflect the daily practice of care for these patients and the successful outcome following PRS injection, as 82% of the patients who reached clinical closure within one year also reached radiological healing within one year. Finally, the composition of the PRS differs between individual patients, which may affect the healing rates [33]. This potential relationship could not be assessed in this study. Ongoing translational and clinical research is needed to study autologous cell therapy as an add-on to surgical treatments, including the standardization of preparation and dose–response relation.

## 5. Conclusions

In this uncontrolled pilot study, additional autologous PRS injection with TAFR showed promising outcomes, as clinical and radiological healing was reached in the vast majority of patients with a (complex) cryptoglandular transsphincteric fistula, who were treated in a tertiary referral center, at long-term follow-up. In addition, the recurrence rates were low. Future randomized research is warranted to study the effects of PRS.

## Figures and Tables

**Table 1:** Baseline characteristics of the study cohort (n = 43).

Variable	Values
Male sex, *n (%)*	30 (70)
Mean age at inclusion (in years), SD	46.1 (12.0)
Median follow-up time (in years), IQR	4.2 (3.5–5.1)
Median duration of symptoms prior to PRS injection (in years), IQR	2.4 (1.1–5.6)
Deviating stoma at time of PRS injection, *n (%)*	3 (7)
Reversal of ostomy during the study period, *n*	1
No wish for reversal, *n*	2
Complex fistula (high transsphincteric and/or multiple side tracts), *n* (%)	38 (88)
Fistula classification, *n* (%) *High transsphincteric* *Low transsphincteric* *Female with an anterior fistula* *Presence of side tracts*	33 (77) 10 (23) 4 (40) 5 (50)
Fistula extensions, *n* (%) *No side tracts* *1 side tract* *≥2 side tracts*	20 (47) 11 (26) 12 (27)
Prior fistula surgery, *n* (%)	41 (95)
Prior fistula procedures aimed at fistula closure, * *n* (%)	22 (56)
Primary clinical closure (<1 year) **, *n* (%)	36 (84)
Radiological healing in patients with primary clinical closure (<1 year) ***, *n* (%)	31 (89)

**Abbreviations.** SD = standard deviation; IQR = interquartile range; PRS = platelet-rich stroma. * Defined as transanal advancement flap repair, ligation of the intersphincteric fistula tract, and/or fistulotomy. ** Clinical closure <1 year without the need for unplanned re-interventions. *** Imaging was obtained in 35/36 patients.

**Table 2:** Overview of unplanned re-interventions (n = 8) during long-term follow-up.

Variable	Value
Number of patients with ≥1 unplanned re-interventions, *n (%)*	5 (12)
Type of re-interventions, *n* Incision and drainage of an abscess Seton placement alone Fistulotomy PRS injection Re-TAFR and PRS injection FiLaC	3 1 1 1 1 1

**Abbreviations**. PRS = platelet-rich stroma; TAFR = transanal flap repair; FiLaC = fistula tract laser closure.

## Data Availability

Requests for the sharing of de-identified data by third parties will, after a written request to the corresponding author, be considered. If the request is approved and a data access agreement is signed, only de-identified data will be shared. Arthrex GMBH: München, Germany, supplied the equipment.
